# Genetic Instability of Influenza pH1N1 Viruses

**DOI:** 10.1128/genomeA.00841-14

**Published:** 2014-10-02

**Authors:** Petri Jalovaara, Dmitrii Bychkov, Laura Ahtiainen, Hannimari Kallio-Kokko, Miia Valkonen, Anu Kantele, Pirkko Mattila, Henrikki Almusa, Olli Kallioniemi, Denis Kainov

**Affiliations:** a The Institute for Molecular Medicine Finland (FIMM), Helsinki, Finland; b Helsinki University Hospital Laboratory, HUSLAB, Helsinki, Finland; c Helsinki University Central Hospital, HUCS, Helsinki, Finland

## Abstract

Here, we report full-length genome sequences of influenza pH1N1 viruses obtained prior to and after propagation in MDCK cells. Paired comparisons of the genomes showed that each strain acquired 1.0 to 18.8 mutations per genome per replication cycle, which corresponds to 0.5 to 5.8 mutations per virus proteome per replication cycle. Our analysis indicates that pH1N1 viruses accumulated adaptive mutations among others in response to propagation in cell culture. These results could be important for vaccine and drug-sensitivity surveillance studies, as well as for vaccine and antiviral drug development programs where cell cultures are used for influenza propagation.

## GENOME ANNOUNCEMENT

Influenza viruses receive much public attention because they cause major pandemics and epidemics heavily impacting public health and economy. Different surveillance, prevention, and treatment programs are under development to improve control of influenza outbreaks. Such programs usually utilize cell culture for virus propagation.

Here, we assess the genome stability of 10 pH1N1 strains prior to and after propagation in MDCK cells. In particular, we sequenced genomes of pH1N1 strains from patient nasopharyngeal aspirates (NPA) and after four passages in MDCK cells as described elsewhere (1, 2). We found that upon propagation, strains acquired 4 to 75 mutations in their genomes (2 to 23 mutations in their proteome). This corresponds to 1.0 to 18.8 mutations per genome per replication cycle (0.5 to 5.8 mutations per proteome per replication cycle).

During propagation in cell culture at least three viruses acquired an N/D239G mutation in HA, an R12K mutation in M2, and N29S and A89V mutations in NEP. Two viruses acquired a V544I mutation in HA, D151E/N, S95N, N386K, K397N, G398E, N449K mutations in NA, D2N, I18V, E55K mutations in NS1, and a V113I mutation in PB1. One virus acquired an interesting E151K mutation in NA.

Mutations E119K and D151E/N in NA have been found previously to be associated with propagation of influenza viruses in cell culture and linked to an oseltamivir-resistant phenotype (2–4). Mutation D/N239G resides in the antigenic site of HA. These mutations can potentially confound serological assays and drug-susceptibility tests commonly used in influenza research.

Interestingly, two influenza pH1N1 strains possess unusual C-terminal extensions of the NS1 protein (230 versus 219 amino acids). Moreover, these viruses retain the extension after passaging in MDCK cells. This indicates that the C-terminal extension and truncation (2) of pH1N1 NS1 do not interfere with the propagation of these viruses in cell culture.

Thus, influenza pH1N1 viruses acquire mutations in their genomes when passaged in cell culture. The dynamic accumulation of mutations allows selection of adaptive mutations. These and other mutations should be taken into consideration by influenza researchers because they could mislead interpretation of drug-sensitivity and antigenicity results and interfere with antiviral drug and vaccine development where virus propagation in cell culture is needed.

### Nucleotide sequence accession numbers.

Twenty whole-genome sequences of 10 pH1N1 isolates from Finland (year 2014) have been deposited in Genbank (accession numbers KM219127 to KM219286).
